# Working mechanisms of Cognitive Behavioral Therapy and Acceptance and Commitment Therapy: a dynamic network approach

**DOI:** 10.1192/j.eurpsy.2024.221

**Published:** 2024-08-27

**Authors:** W. A. Van Eeden

**Affiliations:** Psychiatry, Leiden University Medical Center, Leiden, Netherlands

## Abstract

**Introduction:**

Cognitive Behavioral Therapy (CBT) and Acceptance and Commitment Therapy (ACT) seem be similarly effective for the treatment of major depressive disorder (MDD). However, much remains unknown about the differences in underlying psychological mechanisms of change. Assessing dynamic change of depressive symptoms and treatment-specific psychological constructs over time may yield important insights.

**Objectives:**

The current study will be the first to compare dynamic symptom networks in randomized groups of two psychotherapies by using dynamic time-warp (DTW) analyses.

**Methods:**

We reanalyzed data from a randomized controlled trail of 82 patients suffering from MDD. Three depressive symptom subscales (mood, sleep, appetite/weight) and three treatment-related constructs (dysfunctional attitudes, decentering, and experiential avoidance) were collected at 7 time-points before, during, after treatment, and at up to 12 months follow-up. The DTW-analysis modeled the temporal dynamics of depressive symptoms and treatment-related constructs within each individual after which the findings were aggregated on the group-level. Undirected and directed networks were constructed, of which the latter yielded in- and out-strength for each node, that were compared between treatment arms.

**Results:**

Networks based on symptom and construct dynamics markedly differed between treatment arms. Within the CBT-arm a decrease of experiential avoidance was related to a decrease in dysfunctional attitudes (*d* = 0.059, *p* = 0.008). Within the ACT-arm a decrease of mood symptoms was related to a decrease of experiential avoidance (*d* = 0.051, *p* = 0.04) and an increase of decentering was related to a decrease in sleep symptoms (*d* = 0.038, *p* = 0.02) and appetite/weight symptoms (*d* = 0.049, *p* = 0.03).

**Conclusions:**

DTW offers a promising alternative approach to study and compare working mechanisms of different treatment interventions. Comparing CBT and ACT revealed a decrease in experiential avoidance within CBT and an increase in the ability to decenter within ACT. However, within both treatments a change in other constructs, suggesting that a first alleviation of mood symptoms is important to activate underlying psychological change.

**Disclosure of Interest:**

None Declared

